# A Needs-Based Analysis of Teaching on Vaccinations and COVID-19 in German Medical Schools

**DOI:** 10.3390/vaccines10060975

**Published:** 2022-06-19

**Authors:** Franziska Baessler, Ali Zafar, Katharina Mengler, Ricarda Nadine Natus, Anne Josephine Dutt, Manuel Kuhlmann, Emre Çinkaya, Simon Hennes

**Affiliations:** 1 Centre for Psychosocial Medicine, Department of General Internal and Psychosomatic Medicine, Heidelberg University Hospital, Thibautstraße 4, 69115 Heidelberg, Germany; ali.zafar@med.uni-heidelberg.de (A.Z.); katharina.mengler2@med.uni-heidelberg.de (K.M.); rica@natus-online.de (R.N.N.); a.dutt@t-online.de (A.J.D.); simon.hennes@impf-dich.org (S.H.); 2 Heidelberg Academy of Sciences and Humanities, Karlstraße 4, 69117 Heidelberg, Germany; 3 Impfaufklärung in Deutschland e.V., Simrockstraße 16, 53619 Rheinbreitbach, Germany; manuel.kuhlmann@impf-dich.org (M.K.); emre.cinkaya@stud.uke.uni-hamburg.de (E.Ç.)

**Keywords:** COVID-19, vaccines, vaccination, vaccine education, curriculum mapping, medical curriculum, vaccine hesitancy, vaccine scepticism, teaching, communication

## Abstract

The COVID-19 pandemic highlights the need for improving public confidence in vaccines. Academic gaps and redundancies on vaccinations must be identified to revise the medical curriculum for up-to-date training of medical students. This cross-sectional survey assessed the status of vaccine-related teaching in general and specific to COVID-19 in medical schools across Germany. A total of 4313 medical students completed a questionnaire comprising items on national learning goals and perceived needs for teaching on vaccinations. Mixed methods were used to analyse data quantitatively for relative frequencies (%) and correlations between teaching items and semesters (Spearman’s rho), and qualitatively (content analysis). Our findings showed that 38.92% of the students were dissatisfied with teaching on vaccine-preventable diseases, but the perceived satisfaction increased in later semesters (r = 0.46, *p* < 0.001). Moreover, 75.84% and 68.15% of the students were dissatisfied with teaching related to vaccine scepticism and vaccine-related communication strategies, respectively. Furthermore, 63.79% reported dissatisfaction with teaching on COVID-19 disease and 72.93% with teaching on COVID-19 vaccines. A total of 79.12% stated they educated others on COVID-19 and its vaccines and 75.14% felt responsible to do so. A majority of the medical students were dissatisfied with teaching on dealing with vaccine scepticism, communication strategies and COVID-19 vaccines. We recommend practice-oriented vaccine education, especially for teaching communication skills to medical students.

## 1. Introduction

The global death toll of the COVID-19 pandemic crossed 6 million in March 2022 with 134,606 deaths reported in Germany alone [1,2]. Most countries, including China, where SARS-CoV-2 originated, adopted severe restrictive measures such as lockdowns, physical distancing and border closures to control infections at significant social and economic costs, but only the widespread vaccination of populations restored some normalcy [3,4]. While historically vaccines have controlled infectious diseases and increased life expectancy [5], vaccinations for many preventable diseases remain underutilised [6,7]. Despite the rollout of the first COVID-19 vaccines in December 2020 [8,9], hesitancy among the general population has hampered immunisation efforts in many countries [10,11,12]. In Germany, for instance, the percentage of adults completely against getting vaccinated remains between 12% and 15% [13].

Since physicians are a trusted source of advice on vaccinations for the public [14,15], medical students as future doctors should be well-trained for convincing patients about the usefulness of vaccinations. Previous studies have reported inadequate training at medical schools and the poor communication skills of healthcare workers as major reasons for not recommending vaccinations to patients and parents [16,17,18,19]. A study in France found nearly one-third of final-year medical students inadequately prepared to deal with clinical aspects of vaccination, especially communication issues with vaccine-hesitant patients [20]. During the COVID-19 pandemic, however, medical students expressed a higher willingness to get vaccinated against SARS-CoV-2 than the general population [21,22,23]. These studies suggest that the proper education and training of medical students on vaccinology at school can prove crucial for improving vaccine uptake and controlling future epidemics. Another study conducted in the USA underlines the relevance for curricula designed to improve student expertise and communication skills on vaccines and particularly COVID-19 vaccination [24].

As medical education becomes more and more standardised, curriculum mapping has emerged as a relevant planning tool to plan future instruction and to propose competency-based changes by identifying and addressing knowledge gaps in the curriculum [25,26]. In German medical schools, vaccinology is not offered as a stand-alone subject but is taught within courses such as immunology or virology during the six-year (12 semesters) standard medical curriculum [27,28,29] The national medical education guidelines (Nationaler Kompetenzbasierter Lernzielkatalog Medizin or the NKLM) recommend that schools teach about vaccine-preventable diseases, including the advantages and disadvantages of vaccines, risks and legal bases, and appropriate skills for the counselling of patients and parents [30] However, medical schools implement these guidelines autonomously and the teaching content largely depends on individual lecturers and medical schools. To the best of our knowledge, there has been no systematic evaluation of how or what German medical schools are teaching students about vaccinations in general or COVID-19 vaccines specifically. Therefore, a needs-based assessment can be useful for medical curriculum planners to identify knowledge gaps in the learning outcomes for vaccinations at medical schools.

In this cross-sectional study, we assessed vaccine-related teaching, including COVID-19 vaccines and the corresponding communication skills, based on the learning goals identified in the German national guidelines for physicians. We also determined what and how the students wanted to be taught regarding vaccinations, COVID-19, and communication strategies and their preferences for vaccine education. Additionally, we asked about students’ sense of responsibility and confidence in educating and informing others about vaccines and COVID-19. Our study objective was to highlight the needs of COVID-19-related teaching and vaccinations in general from the point of view of medical students. These findings will be helpful in understanding and addressing knowledge gaps in teaching about vaccinations at medical schools and provide the groundwork for evidence-based changes in the curriculum.

## 2. Materials and Methods

### 2.1. Study Participants 

Primary inclusion criteria for participation were enrolment at a medical school in Germany and age of consent (≥18 years). Exclusion criteria were not being a medical student, being a dental medicine student (since they study a different curriculum) and a lack of age of consent (<18 years). 

All state-run medical schools in Germany (n = 38) were approached to distribute an online questionnaire in the faculty to recruit enrolled students. Students from all semesters were eligible to participate. There were approximately 100,000 students enrolled in medical schools across Germany with female students comprising over two-thirds of them [31,32]. Since proficiency in the German language is a prerequisite for admission to medical schools in Germany, the survey was conducted in the German language. Although all public medical schools were contacted, data privacy requirements about the identification of schools and individual students prevents us from identifying exactly how and which schools distributed the questionnaire.

### 2.2. Survey Instrument 

A questionnaire comprising items on vaccine readiness, uptake and risk assessment/perception, risk of infection, participation in education/informing about vaccination and demographic data was derived partially from previously validated instruments such as the 5C model of vaccination readiness [33,34]. A separate smaller section with 19 questions specifically on vaccine-related teaching was developed based primarily on the German national guidelines for physicians’ recommendations for vaccination-related learning outcomes [30]. These guidelines describe the framework of the professional competencies for qualified medical graduates. Several basic learning goals on vaccine education in the NKLM were included in the questionnaire to design items on vaccine-preventable diseases, the advantages and side effects of vaccines, risks and legal bases, and appropriate skills for the counselling of patients and parents. Similar approaches for developing questionnaires from the NKLM have been used in earlier studies [35,36].

Participants were asked about the status of teaching on vaccinations at their school, different teaching methods used and their opinion about the most needed aspects of vaccine education on a 7-point Likert-like scale. Only the endpoints of the scale (from 1 = not agree at all to 7 = fully agree) were labelled, but each point was assigned to the degree of disagreement or agreement to the statement.

As medical students are often asked about health-related topics by the people around them (peers, family/friends, patients, etc.), we also assessed to what extent medical students educated and informed others about COVID-19 and felt responsible for doing so. Therefore, additional variables from an earlier section were included in the analysis with the objective of suggesting improvements in the curriculum with respect to the timing of teaching certain topics.

One open-ended question asked “How could teaching about vaccine education and vaccinations be further improved?” The participants were asked to comment in detail about the vaccine-related content needed in the medical curriculum and to make suggestions on how to improve vaccine education at schools. 

The online link (via www.unipark.com) (3 March 2021) to complete the questionnaire was accessible from 30 March until 18 April 2021.

### 2.3. Data Analysis

All scores as well as demographic data were evaluated descriptively. Answering demographic and some baseline items was mandatory for continuing with the questionnaire. All questions related to teaching on vaccinations were optional to answer, resulting in different response numbers for each question. The missing values were excluded from analysis and results were reported only for the participants who answered the individual items. Questionnaires completed in less than 5 min were excluded from data analysis over reliability concerns. One completed questionnaire was excluded for stating the participant’s age was above 80 years. Quantitative data were analysed by R.

For better readability of results, the first three points (1–3, <4) on the 7-point scale were interpreted as disagreement with the statement (dissatisfaction with teaching) and the last three points (5–7, >4) were interpreted as agreement with the statement (satisfaction with teaching). The cumulative percentages of students choosing answers between 1 and 3 or 5 and 7 for an individual item on teaching were interpreted as dissatisfied or satisfied students, respectively. Additionally, the translated questionnaire is provided in the Supplementary Materials.

Similarly, agreement and disagreement with items on teaching needs and wishes were interpreted as endorsement for more or less content on the topic in the curriculum. Moreover, each item related to teaching was correlated individually with the semester. Spearman’s rank order correlation (Spearman’s rho, r_s_ > 0.10, small correlation; r_s_ > 0.30, moderate correlation; r_s_ > 0.50, large correlation) was used because the items were ordinal scaled and not normally distributed [37].

Free-text answers to the question “How can teaching about vaccine education and vaccinations be further improved?” were analysed via qualitative content analysis [38]. The coding process was carried out by RN (psychology) using inductive category formation from repetitive sequences to discover thematic categories. All responses were evaluated line by line to generate a coding guide. Two authors (SH and EC) revised the codes and the final categories were agreed upon after consensus (inter-rater reliability = 76%).

Since practical-year students work full-time in hospitals, often with close patient contact, the data of these participants were identified separately because of the special interest in different demands and status for teaching.

## 3. Results

**Figure 1:**
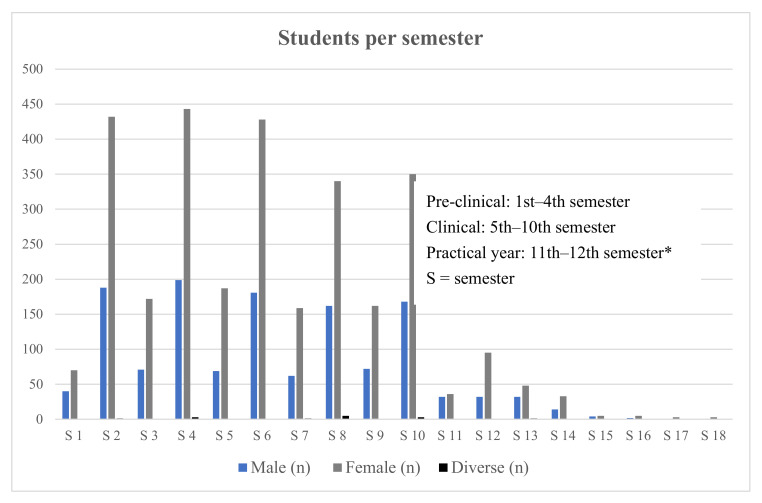
Distribution of medical students enrolled per semester. * Six-year undergraduate medical education in Germany comprises two pre-clinical years and a four-year clinical segment ending with a final practical year. Some students take more time to complete their studies than the official minimum duration.

Overall, 4313 (female = 2971/68.88%, male = 1328/30.79%, diverse = 14/0.32%) participants fit the selection criteria. The mean age was 24.03 years (SD = 3.85). The sample population was distributed over 18 semesters with 94.34% (n = 4069) studying in pre-clinical/clinical years and 5.66% (n = 244) in their practical year (Figure 1). About 1.74% (n = 75) of the participants were engaged in a vaccination education initiative at the time of the survey.

### 3.1. Vaccine Education in General

**Figure 2:**
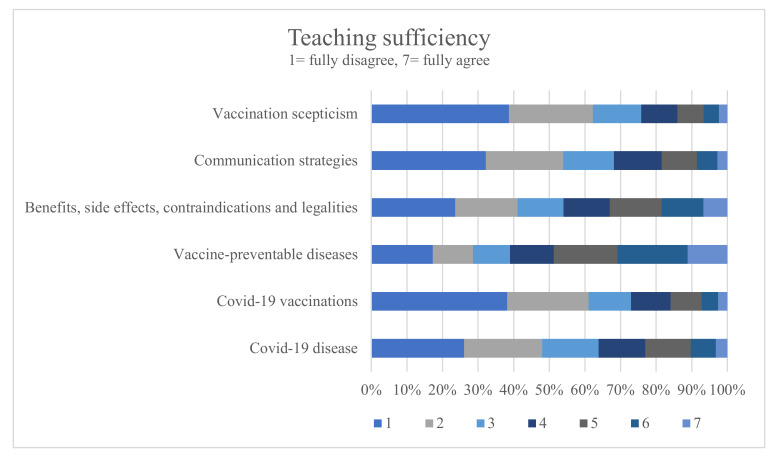
Percentage agreement to statements regarding teaching of vaccination-related topics.

Overall, 48.8% of the students rated the teaching on “vaccine-preventable diseases” to be sufficient. Perceived satisfaction increased among the students in later semesters (r = 0.46, *p* < 0.001). Figure 2 illustrates the results on perceived satisfaction with a teaching topic.

The majority of the practical-year students (85.16%) were satisfied with teaching on “vaccine-preventable diseases”. In total, 33.05% of the students rated teaching on “benefits, side effects, risk, contraindications and legal implications of vaccines” as sufficient. The perceived satisfaction increased in higher semesters (r = 0.36, *p* < 0.001). A total of 64.07% of the practical-year students stated they were satisfied with teaching on “benefits, side effects, risk, contraindications and legal implications of vaccines”. Missing values differed slightly (239–342) between the teaching-related items (Table 1).

The least number of students rated teaching as sufficient on vaccine scepticism (14.02%) and on communication strategies for vaccination counselling (18.45%). Dissatisfaction could be shown especially for the earliest semesters. Perceived satisfaction with teaching on vaccination-related communication skills increased in higher semesters (r = 0.22, *p* < 0.001). Still, only 34.05% of the practical-year students were satisfied with the teaching of communication skills. Teaching on the topic of vaccinations was deemed important by 97.01% of the students while 86.39% of the students endorsed more teaching on “benefits, side effects, risk, contradictions and legal implications of vaccines”; 83.76% on “vaccination counselling”; and 85.33% of the students endorsed more teaching on dealing with “vaccine scepticism”. Most of the participants endorsed more practice-oriented teaching (78.67%) and practical exercises (65.56%) such as role play and simulated patients. A total of 81.62% of the students were in favour of teaching skills to induce a “pro-vaccine” attitude.

**Figure 3:**
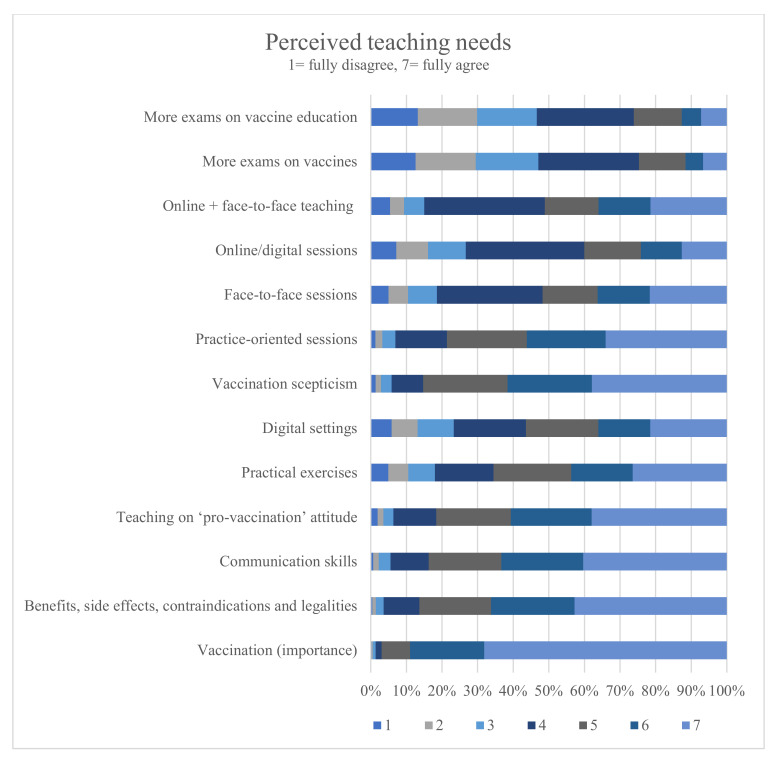
Percentage agreement to statements regarding perceived needs of students on vaccine-related teaching topics.

Figure 3 illustrates the results on perceived teaching needs of a given topic.

### 3.2. COVID-19 Related Teaching

Overall, 23.08% of the participants rated teaching on COVID-19 disease and COVID-19 vaccines (15.97%) as sufficient. Fewer practical-year students reported sufficient teaching on COVID-19 (15.63%) and COVID-19 vaccines (9%). 

The majority of students (79.12%) stated they educated others about COVID-19 and its vaccines and also felt responsible to do so (75.14%) and the tendency for agreement with both statements increased in higher semesters (r = 0.14, *p* < 0.001 and r = 0.12, *p* < 0.001, respectively). Most students in their practical year agreed with the corresponding statements (83.95% and 78.18%, respectively).

**Figure 4:**
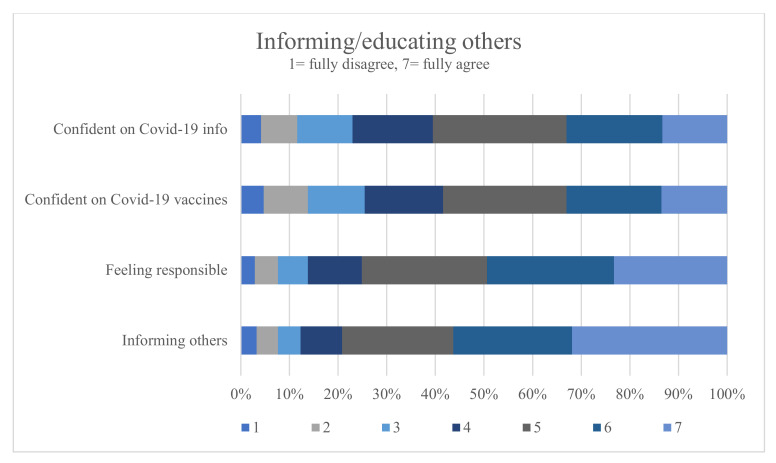
Percentage agreement of questions about informing/educating others and confidence about the learnt information.

Fewer students agreed with the statement that they were confident in answering questions from patients about COVID-19 (60.51%) and its vaccines (58.4%) (Figure 4). The correlation with being confident in answering questions about COVID-19 and its vaccines increased in later semesters (r = 0.18, *p* < 0.001). Practical-year students stated that they were more confident in answering questions about COVID-19 (70.37%) and COVID-19 vaccines (74.48%).

### 3.3. Student-Suggested Improvements

In the qualitative analysis of open-text answers (n = 248), several major needs were identified by the students related to: (a) improvement in content and (b) improvement of formats. Where suitable, subcategories were identified from the answers for each category (Supplementary Materials). The results are shown in the order of the frequencies of the codes being mentioned. Examples of quotes are included to illustrate the suggestions.

The most frequently mentioned learning goal by the students was for better training on communicative strategies to deal with vaccine scepticism or sceptics (UI01). The students demanded more teaching on the topic of vaccinations to be integrated into the medical curriculum (BD02), specifically mentioning the topic of teaching about the benefits and side-effects of vaccines (UI04) for adequate counselling:

1. “…more in-depth discussion of the risks of vaccinations in order to meet people with scepticism at eye level and to be able to argue according to their fear.”—6th semester student

The students suggested giving more relevance to practical aspects of teaching (MP). They asked for more practice-oriented content in the curriculum such as practicing counselling with simulated patients (MP01 and MP03) in tandem with learning conversation techniques for dealing with vaccine opponents/sceptics (UI01):

1. “Role-playing with vaccine opponents or vaccination sceptics in several levels with regard to their scepticism.”—10th semester student
2. “Simulate patient interviews, work out pros and cons, communicate vaccines with names, possible side effects and benefits…”—8th semester student

The students were also dissatisfied with a lack of teaching content on the COVID-19 pandemic (C03) and its vaccinations:

1. “…more education and teaching should be made possible here so that we can also contribute a part to educating the population. Because I couldn’t better answer such questions that were put to me.”—8th semester student

Another recurring theme was the need for teaching skills on dealing and communicating scientific knowledge and evidence-based medicine (MF02):

1. “Most important: understanding scientific content in order to be able to explain it in simple terms and to the point when talking to patients/acquaintances/relatives.”—6th semester student
2. “More information on how to make patients aware of the statistics so that they do not only think about whether they might be the 1% who suffer severe side effects. How to educate in a time-efficient way without losing information. (In the vaccination centre, this is supposed to happen in 3 min).”—6th semester student

## 4. Discussion

The topic of vaccinations has fuelled intense debates around the world during the COVID-19 pandemic. This study aimed to assess the teaching content on vaccine education in general and specifically on COVID-19 in an effort to identify gaps in the medical curriculum and its implementation. The triangulation of quantitative and qualitative results showed that a majority of medical students were dissatisfied with teaching related to most aspects of vaccinations, although the perceived satisfaction improved in the later semesters. 

### 4.1. Vaccine Education in General

The majority of students rated the status of teaching on all aspects of vaccinations, including COVID-19, insufficient, suggesting a general dissatisfaction within the medical curriculum. These findings are similar to several recent studies that have reported on significant gaps in the knowledge of medical students regarding vaccinations [20,39]. More students in higher semesters were satisfied with vaccine-related teaching except for COVID-19. The slight increase in student satisfaction in higher semesters suggests that vaccinology topics are taught more in the later semesters. Vaccinology is not offered as an exclusive course at German medical schools but rather within subjects such as microbiology, infectiology and virology. For instance, the Heidelberg Medical School teaches basic principles of vaccines during the 2nd semester but this does not cover aspects of vaccine counselling. More specific topics are taught in the 5th semester with virology, where scepticism and conspiracy are discussed using the example of measles. This is primarily because students can only absorb a certain amount of information from a large amount of exam-related, fact-based knowledge in the preclinical semesters. Moreover, vaccinology may not be taught without establishing prior knowledge in molecular biology, immunology and infectiology. 

The learning goal with the highest number of satisfied students (48.8%) was “vaccine-preventable diseases”, which also increased in later semesters (r = 0.46). Apart from the ongoing unprecedented context of COVID-19, this finding is in line with the implementation of the NKLM in Germany [30]. The topics described in detail in the guidelines, such as vaccine-preventable diseases and the pros and cons of vaccinations, were rated to be better taught than topics mentioned briefly (if at all), such as vaccine scepticism. The NKLM was revised in April 2021 and now accords more importance to communication of vaccine-related information, especially a participatory approach in consulting with patients [40]. However, a presumptive approach or motivational interviewing has shown more promising results [41,42,43]. It remains to be seen how schools and teachers in Germany incorporate the latest recommendations, warranting further curriculum mapping studies.

### 4.2. COVID-19-Related Teaching

Our findings showed that only 15.63% of the practical-year students were satisfied with teaching regarding the COVID-19 disease. Students in their final year regularly work under supervision in the hospital wards and might have experienced less sufficient teaching because they were often involved in working with patients and perceived a higher responsibility to help others during the pandemic. This survey was conducted in April 2021, more than one year after the German government imposed a national lockdown and medical schools were forced to shift teaching sessions to online platforms [44,45,46]. Since medical curricula are normally set years ahead, our findings highlight the challenge faced by medical schools and teachers in rapidly integrating new content and schedules to incorporate emerging changes in disease epidemiology of a global significance, possibly because of the significant additional demands put on them under pandemic-related restrictions. During the pandemic, university hospitals and medical faculties should have been given adequate resources to prepare future doctors for the upcoming challenges.

While dealing with vaccine scepticism was relevant before the COVID-19 pandemic, a lack of training and proficiency among medical students was noticeable during the crisis and in this study. This suggests a pressing need for improving communication skills teaching for vaccine counselling. The World Health Organisation recognised vaccine hesitancy among the top 10 global health threats in 2019 [47]. The hesitancy debates will only become even more prevalent as countries struggle to convince people to get vaccinated voluntarily. Previous studies show that healthcare providers are uncomfortable with discussing misconceptions about the adverse effects of vaccines with their patients and admit to being unsure about relationships between vaccines and certain diseases [48,49]. By relying on evidence on the safety and benefits of vaccines and properly endorsing vaccines using appropriate communication skills, well-trained physicians as well as medical students can play a crucial role in combating vaccine hesitancy by having productive conversations and building trust with vaccine-hesitant families and individuals.

### 4.3. COVID-19 Vaccinations 

Medical students are often consulted by family, friends and patients for health-related advice. The majority of students from all semesters stated that they informed others about COVID-19 and its vaccines and felt the responsibility to educate others. This tendency increased among students in later semesters. However, only 15.97% of all the students and 9% of the practical-year students rated teaching on COVID-19 vaccines to be sufficient. A high motivation to educate themselves might explain why many more students felt confident to educate others and stated doing so. Additionally, as their studies progress, students gain factual knowledge and clinical training that helps them to gain more confidence. Accompanied with practical work, patient contact becomes more frequent and counselling and educating patients becomes a routine part of their education. However, our data showed that students in earlier semesters also perceived a lack of teaching on communicative skills for vaccination education and counselling. The gap in teaching was further highlighted by the differences between felt responsibility and perceived confidence to educate others and patients throughout all semesters. To overcome these barriers and fulfil the needs for enabling students to adequately inform others, it would be advisable to include teaching on vaccines and communication skills specifically to deal with vaccine sceptics from early on in the curriculum. 

### 4.4. Student-Suggested Improvements

A majority of our participants agreed with the need for practice-oriented teaching. Many suggested interactive teaching sessions for vaccine education, such as role play and simulated patients. Studies have shown that practical teaching is more effective than lectures in facilitating the learning process and improving knowledge outputs [20,50,51,52]. In one needs-assessment study, students enjoyed learning from flipped classroom and case-based formats, and felt more confident in their approach towards vaccine-related discussions [53]. Teaching with the help of role-play and simulated patients also has a positive effect on medical students’ interpersonal communication skills that are useful in medical consultations [54]. 

The participants’ endorsement for interactive, practical teaching involving role plays and simulated patients shows a potential approach for filling knowledge gaps. A simple solution could be to offer an easily accessible elective course on the topic of vaccines and vaccination in the medical curriculum. Elective courses complement the core curriculum by providing students with an opportunity to gain knowledge outside of the routine syllabus and can stimulate “autonomous motivation” among them [55,56]. They also give students a chance to personalise the curricula for a better a learning experience and academic success [57,58]. 

In line with previous findings our study identified students with a special interest in educating the public about vaccinations. Their motivation and knowledge could be utilised to improve the medical curriculum. Peer-teaching and role play may be advisable approaches. In several countries, peer teaching at undergraduate medical schools has been encouraged as part of the formal curriculum to help students train as educators and supervisors in anticipation of their roles as future doctors [50].

## 5. Limitations

Like most online self-administered surveys, not all questions were answered in our questionnaire, leading to differences in n-values for individual items. For ensuring transparency, we have provided the complete case analysis of all included answers along with the missing values in Table 1. Though only 4313 of about 100,000 students enrolled in German medical schools participated in the survey, the large sample size is representative and exceeds the number of participants of a meta-analysis from 2022 that evaluated the prevalence of COVID-19 vaccination among medical students worldwide [59]. The gender ratio was in favour of female students by 70 to 30. However, this proportion corresponds to the current student population in medical schools in Germany [32]. About 1.7% of our participants were involved with an educational initiative on improving vaccination uptake in Germany, which might be an overrepresentation. Though accounting for only 75 participants, this selection/recruitment bias should be considered when interpreting the findings because of their higher motivation to participate in this study. 

Moreover, results about a lack of teaching on vaccinations should be interpreted with caution considering our approach based on self-assessment by students and the pressure on both the teaching staff and students during the pandemic. However, our findings support previous studies that have pointed out a lack of teaching on the topic of vaccinations at medical schools in several countries [20,28,60,61], underlining the need to either include vaccine-related topics in the courses offered in the earlier semester or to introduce a separate well-designed vaccinology course as recommended in earlier studies [62,63].

## 6. Conclusions

During the COVID-19 pandemic, the topic of vaccinations has gained critical importance amidst a growing global anti-vaccination movement. Since physicians play a primary role in the vaccination and education of the population, this study was conducted to assess the training status and needs of medical students, who as future healthcare providers will be convincing their patients and the public about the usefulness of vaccinations. Our results showed a low perceived status of teaching on the topic of vaccinations in general among students in Germany, especially in the earlier semesters. The results provided strong endorsement for giving more importance to communication topics and identified a major gap in teaching about dealing with vaccination scepticism. Medical students reported widespread dissatisfaction with teaching related to COVID-19 vaccines in particular. The students desired integration of more vaccine-related content in the medical curriculum, particularly on communication strategies. They were in favour of practice-oriented vaccine education with suggestions for practical sessions with role play or simulated patients. Interactive, practical teaching with the use of role-plays and simulated patients either as elective courses or as a compulsory part of the medical curriculum are advised as potential solutions and teaching approaches.

We aimed to highlight the needs of teaching about vaccinations in general as well as COVID-19 from the point of view of medical students. Our findings contribute towards acknowledging and bridging the learning gap in vaccine education, considering the insufficient knowledge of vaccination topics among healthcare providers. In order to improve content structure about vaccine-related teaching, further needs assessment studies from the perspective of teachers and other stakeholders in medical curriculum planning as well as knowledge and skills based assessment studies should be conducted to obtain a broader consensus for creating a curriculum map in line with the German national education guidelines. Our results can help to prepare and train the medical students in their role as (future) doctors and facilitate the positive influence they can have on patients’ vaccination decisions by knowing how to talk about vaccines and counter vaccine hesitancy.

## Figures and Tables

**Table 1:** Complete case analysis of questions related to teaching status on vaccinations and informing others, shown for all students who answered the individual items. Relative percentages are shown of participants choosing individual values on a 7-point Likert-like scale: only the endpoints 1 (fully disagree) and 7 (fully agree) were labelled in the questionnaire.

											Correlation with Higher Semesters	
Item	N (4313)	Missing Values	1 (%)	2 (%)	3 (%)	4 (%)	5 (%)	6 (%)	7 (%)	Median	r_s_	*p*-Value
**Teaching sufficiency**												
COVID-19	4063	250	26.04	21.95	15.80	13.12	12.80	7.06	3.22	2	0.02	0.33
COVID-19 vaccinations	4057	256	38.13	22.85	11.95	11.09	8.75	4.61	2.61	2	−0.04	0.009
Vaccine-preventable diseases	4041	272	17.27	11.23	10.42	12.27	17.92	19.72	11.16	4	0.46	<0.001
Benefits, side effects, contraindications and legalities	4045	268	23.56	17.53	12.86	13.00	14.56	11.72	6.77	3	0.36	<0.001
Communication strategies	4025	288	32.15	21.69	14.31	13.39	9.96	5.71	2.78	2	0.22	<0.001
Vaccination scepticism	4028	285	38.63	23.61	13.60	10.13	7.35	4.29	2.38	2	0.13	<0.001
**Perceived teaching needs**												
Vaccination (importance)	4074	239	0.22	0.32	0.83	1.62	7.98	20.89	68.14	7	0.02	0.27
Benefits, side effects, contraindications and legalities	4051	262	0.39	1.06	2.15	10.00	20.19	23.45	42.75	6	0.04	0.009
Communication skills	4040	273	0.69	1.51	3.32	10.72	20.42	22.97	40.37	6	0.03	0.09
Teaching on “pro-vaccination” attitude	4019	294	1.89	1.67	2.81	12.02	20.93	22.72	37.97	6	0.04	0.01
Practical exercises	4030	283	4.91	5.58	7.47	16.48	21.86	17.27	26.43	5	−0.01	0.69
Digital settings	4008	305	5.81	7.34	10.13	20.28	20.31	14.57	21.56	5	−0.03	0.03
Vaccination scepticism	4021	292	1.32	1.54	2.96	8.85	23.70	23.73	37.90	6	0.02	0.19
Practice-oriented sessions	3999	314	1.28	1.90	3.73	14.43	22.48	22.16	34.03	6	0.02	0.21
Face-to-face sessions	3989	324	4.96	5.52	8.05	29.71	15.47	14.67	21.63	4	0.04	0.008
Online/digital sessions	3990	323	7.14	8.92	10.58	33.28	15.99	11.43	12.66	4	0.03	0.07
Online + face-to-face teaching	3976	337	5.43	3.90	5.63	33.88	15.17	14.49	21.50	4	0.06	<0.001
More exams on vaccines	3971	342	12.59	16.87	17.58	28.25	13.07	4.91	6.72	3	0.03	0.07
More exams on vaccine education	3976	337	13.20	16.65	16.75	27.31	13.43	5.41	7.24	3	0.01	0.72
**Informing/educating others**												
Informing others	4302	11	3.23	4.35	4.70	8.55	22.87	24.41	31.89	6	0.14	<0.001
Feeling responsible	4300	13	2.88	4.70	6.21	11.07	25.74	26.14	23.26	5	0.12	<0.001
Confident in COVID-19 vaccines	4296	17	4.66	9.12	11.66	16.15	25.35	19.53	13.52	5	0.18	<0.001
Confident in COVID-19 info	4290	23	4.13	7.46	11.35	16.55	27.46	19.72	13.33	5	0.18	<0.001

## Data Availability

The anonymised dataset generated and analysed during this study has been made available by the corresponding author in the Supplementary Materials of the manuscript.

## Supplementary Materials

The following supporting information can be downloaded at: https://www.mdpi.com/article/10.3390/vaccines10060975/s1, File S1: The questionnaire used for the study is provided in File S1 and can be used for research purposes given that proper reference is provided to the original paper and authors. File S2: The coding methodology used for the analysis of qualitative data is provided in File S2. Table S1: The dataset associated with the analysis and results for this study is provided in Table S1.
