# Supplementary material for: A Needs-Based Analysis of Teaching on Vaccinations and COVID-19 in German Medical Schools
Source: Vaccines (Basel). 2022 Jun 19;10(6):975. doi: 10.3390/vaccines10060975 (PMC9228741; doi:10.3390/vaccines10060975)
Supplement: Supplementary file 1 [file vaccines-10-00975-s001.zip › File S1.pdf]

German to English translated non-demographic, questionnaire/items included in this study

**1. To what extent were you taught the following in medical school** … was taught sufficiently”	1. (agree) not at all …..................................... 7. (agree) fully						
COVID-19 disease…	◻	◻	◻	◻	◻	◻	◻
COVID-19 vaccinations…	◻	◻	◻	◻	◻	◻	◻
Vaccine-preventable diseases…	◻	◻	◻	◻	◻	◻	◻
Benefits, side effects, risks, contraindications and legal foundations of vaccinations…	◻	◻	◻	◻	◻	◻	◻
Adequate communication strategies for vaccination education and risk communication…	◻	◻	◻	◻	◻	◻	◻
Dealing with vaccination scepticism…	◻	◻	◻	◻	◻	◻	◻

**2. How much do you agree with the following statements about teaching:**	1. (agree) not at all …..................................... 7. (agree) fully						
Teaching about vaccination is important.	◻	◻	◻	◻	◻	◻	◻
Teaching about the benefits, side effects, risks, contraindications and legal foundations of vaccinations should be given more weight.	◻	◻	◻	◻	◻	◻	◻
Teaching of skills for the doctor-patient-conversation regarding vaccination education/counselling should be given more weight.	◻	◻	◻	◻	◻	◻	◻
Teaching of skills to generally bring about a “pro-vaccination decision” should be given more weight.	◻	◻	◻	◻	◻	◻	◻

Practical exercises (e.g. role-plays with patients) on vaccination education should be given more weight.	◻	◻	◻	◻	◻	◻	◻
Teaching about educational conversations in digital settings (e.g., video chats) should be given more weight.	◻	◻	◻	◻	◻	◻	◻
Teaching about dealing with vaccination scepticism should be given more weight.	◻	◻	◻	◻	◻	◻	◻
Teaching on the topic of vaccination should be mediated more practice-oriented.	◻	◻	◻	◻	◻	◻	◻
Teaching on the topic of vaccination should be done in face-to-face sessions.	◻	◻	◻	◻	◻	◻	◻
Teaching on the topic of vaccination should be done online/digitally.	◻	◻	◻	◻	◻	◻	◻
Teaching on the topic of vaccination should be done in a mixture of online and face-to-face sessions.	◻	◻	◻	◻	◻	◻	◻
Examinations on the topic of vaccination should be given more weight.	◻	◻	◻	◻	◻	◻	◻
Examinations on the topic of vaccination education should be given more weight.	◻	◻	◻	◻	◻	◻	◻

**3. How much do you agree with the following statements about teaching?**	1. (agree) not at all …..................................... 7. (agree) fully						
I feel responsible for educating other people about COVID-19 and the vaccines.	◻	◻	◻	◻	◻	◻	◻
I educate other people (e.g., family, friends) about COVID-19 and the vaccines.	◻	◻	◻	◻	◻	◻	◻
I am confident that I can answer questions from patients about COVID-19 vaccinations.	◻	◻	◻	◻	◻	◻	◻

I am confident that I can answer questions from patients about the disease COVID-19.	◻	◻	◻	◻	◻	◻	◻

**4. What could be done to further improve teaching on the topic of 'vaccination' and 'vaccination education'? Would you like to write us another comment?**
