# Supplementary material for: A Needs-Based Analysis of Teaching on Vaccinations and COVID-19 in German Medical Schools
Source: Vaccines (Basel). 2022 Jun 19;10(6):975. doi: 10.3390/vaccines10060975 (PMC9228741; doi:10.3390/vaccines10060975)
Supplement: Supplementary file 1 [file vaccines-10-00975-s001.zip › File S2.pdf]

Coding Guide

How could teaching about vaccination education and immunization be further improved?

Approach:

- Qualitative content analysis
- Inductive category formation
- n = 248

Thematic overview map

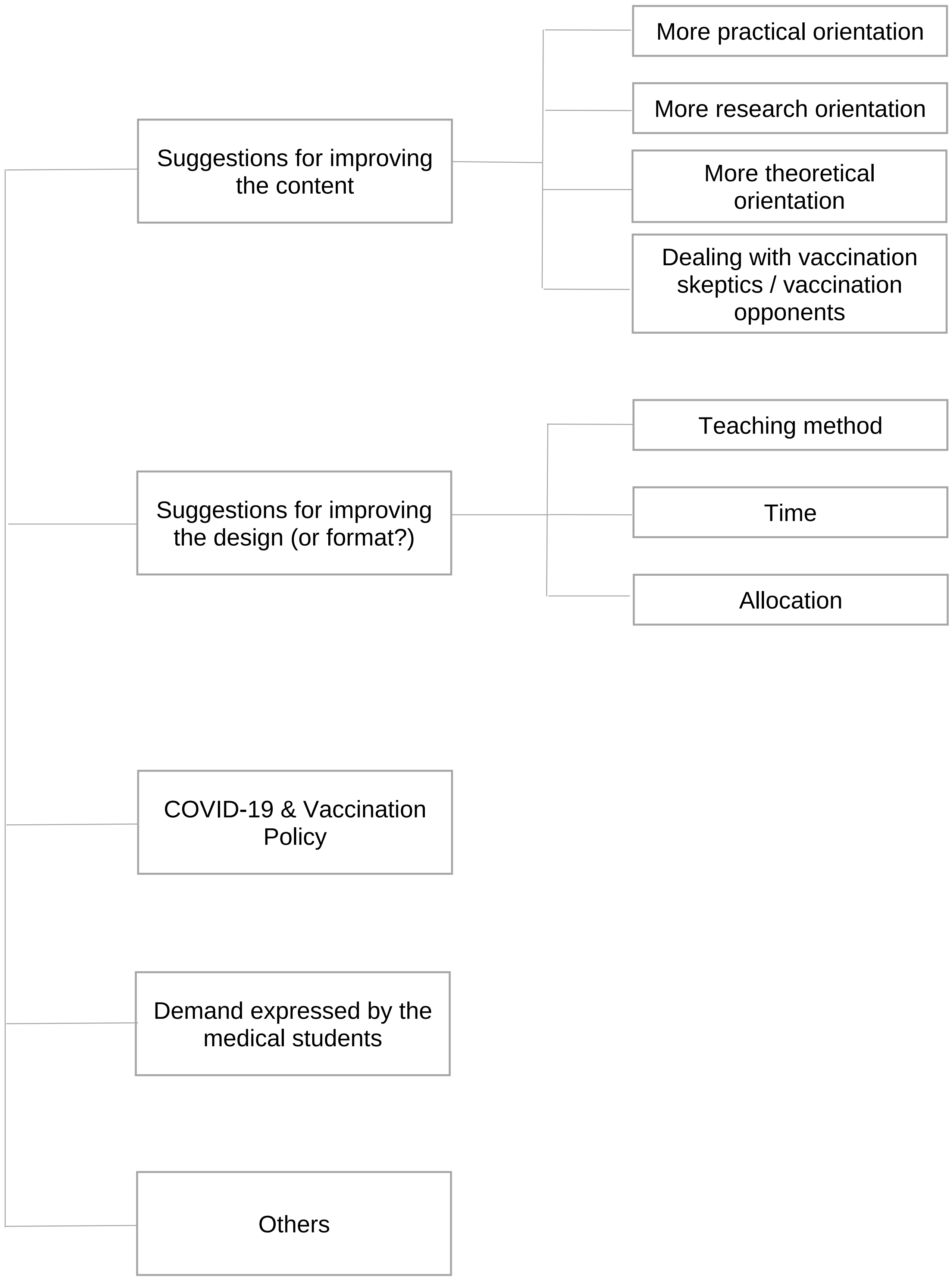

Suggestions for improving the content

	Coding	Quantity
**More practical orientation**	Total	48
Practical exercises (not specified)	MP02	12
Simulation patients / role plays	MP01	11
Vaccination education / Consultation	MP03	10
Practice vaccination	MP08	8
Case studies from clinical practice	MP06	6
Excursion to a vaccine manufacturer	MP07	1

	Coding	Quantity
**More research orientation**	Total	21
Dealing with epidemiological and scientific findings	MF02	18
Recommended reading	MF03	2
Abmoss article	MF04	1

	Coding	Quantity
**More theoretical orientation**	Total	27
Background knowledge on vaccination, vaccines, vaccine commission, etc.	MT01	15
Recognizing misinformation	MT03	8
Ethics and immunization education	MT04	3
Vaccination in relation to general practitioners / Immunization knowledge as it relates to family practice.	MT02	1

	Coding	Quantity
**Dealing with vaccination skeptics / vaccination opponents**	Total	82
Conducting conversations with vaccination skeptics / vaccination opponents	UI01	42
Pro and contra vaccination	UI04	26
Vaccination as a free decision	UI02	8
Discussion sessions, e.g. with vaccination skeptics	UI03	6

Formale Verbesserungsvorschläge der Medizinstudierenden

	Coding	Quantity
**Teaching method**	Total	35
As an elective course offering	LM03	9
As interactive teaching formats e.g. seminar, POL, etc.	LM12	8
As a lecture	LM02	6
Up-to-date and available (learning) materials, e.g. via the teaching platform Moodle	LM13	5
No additional examination	LM05	4

Classroom teaching	LM07	2
Physicians from clinical practice as lecturers	LM10	1

	Coding	Quantity
**Time**	Total	8
To be offered in the first semesters of study	ZP01	3
To be offered continuously in the course of studies	ZP03	4
To be offered in the higher semesters	ZP02	1

	Coding	Quantity
**Allocation**	Total	12
Provide immunization/vaccination education across disciplines.	ZU01	5
With reference to interviewing Gesprächsführung?	ZU03	3
With reference to general medicine	ZU02	2
With reference to microbiology/virology	ZU04	2

COVID-19 & Vaccination Policy

	Coding	Quantity
**COVID-19 & Vaccination Policy**	Total	46
Criticism of teaching on COVID-19	C03	25
Criticism of vaccination campaign in Germany (general and specific to COVID-19)	C02	9
Criticism of vaccine distribution to students	C04	4
Promotion of student projects	WI04	4
Vaccination education in schools	S05	4

Demand expressed by the medical students

	Coding	Quantity
**Demand expressed by the medical students**	Total	48
Wish to integrate immunization/vaccine education into the curriculum	BD02	38
Satisfied with current teaching on immunization	BD03	7
No interest in more teaching on immunization/vaccine education	BD01	3

Others

	Coding	Quantity
**Others**	Total	44
Student has not yet attended a course on immunization/vaccine education and does not indicate whether it will be offered during the course of study	S04	21
Not usable	S02	12

Course on vaccination/vaccination education is only offered later in the course of study.	S03	6
So far not part of the medical studies	S01	5

*Relevant categories for the results section > 10
